# The Tensile, Thermal and Flame-Retardant Properties of Polyetherimide and Polyetherketoneketone Processed via Fused Filament Fabrication

**DOI:** 10.3390/polym16030336

**Published:** 2024-01-26

**Authors:** Tatjana Glaskova-Kuzmina, Didzis Dejus, Jānis Jātnieks, Elīna Vīndedze, Irina Bute, Jevgenijs Sevcenko, Andrey Aniskevich, Stanislav Stankevich, Behnam Boobani

**Affiliations:** 1 Baltic3D.eu, Braslas 22D, LV-1035 Riga, Latvia; didzis@baltic3d.eu (D.D.); janis@baltic3d.eu (J.J.); elina@am-craft.com (E.V.); 2 Institute for Mechanics of Materials, University of Latvia, Jelgavas 3, LV-1004 Riga, Latvia; irina.bute@lu.lv (I.B.); jevgenijs.sevcenko@lu.lv (J.S.); andrejs.aniskevics@lu.lv (A.A.); stanislavs.stankevics@lu.lv (S.S.); 3 Latvian Academy of Sport Education, Brivibas 333, LV-1006 Riga, Latvia; behnam.boobani@lspa.lv

**Keywords:** tensile properties, thermal properties, flame retardancy, fused filament fabrication, anisotropy, polyetherimide, polyetherketoneketone

## Abstract

Polymer materials are increasingly widely used in high-fire-risk applications, such as aviation interior components. This study aimed to compare the tensile, thermal, and flame-retardant properties of test samples made from ultra-performance materials, polyetherimide (PEI) and polyetherketoneketone (PEKK), using the fused filament fabrication process (FFF). The tensile tests were performed for these materials at different raster angles (0, 45, and 90°). The thermomechanical tests were done in the axial, perpendicular, and through-thickness directions to the extruded filaments. The impact of printing parameters on the flame retardancy of 3D-printed samples was investigated in vertical burn tests with varying specimen thicknesses and printing directions. Experimentally, it was testified that PEKK had better isotropic behaviour than PEI for mechanical performance, thermal expansion, and fire-resistant properties, which are essential in fabricating intricately shaped products.

## 1. Introduction

Additive manufacturing has gained widespread adoption across various sectors due to several compelling motivations, such as design flexibility, reduced material waste, rapid prototyping, customization, supply chain simplification, tooling and assembly reduction, and environmental benefits [1,2,3]. These advantages drive the use of additive manufacturing across various industries, e.g., aerospace, automotive, healthcare, defence, and military, promising improved efficiency, reduced costs, and innovative product development [4,5].

Current 3D printing technologies encompass diverse additive manufacturing processes that create three-dimensional objects by adding material layer by layer. The most used 3D printing technologies for producing polymer parts are Fused Deposition Modeling (FDM) or Fused Filament Fabrication (FFF), which are often used interchangeably with stereolithography (SLA) and Selective Laser Sintering (SLS). These 3D printing technologies have strengths, limitations, and specific applications, making them suitable for various industries and use cases [6,7]. Technology selection depends on material requirements, precision, speed, and budget [8].

Three-dimensional printing has gained significant importance in aviation applications due to its capacity to prototype and manufacture complex, lightweight components rapidly. Various 3D polymer materials are used in aerospace to meet the industry’s stringent requirements, including high strength-to-weight ratios, resistance to extreme temperatures, and fire resistance [9]. The commonly used 3D polymer materials for aerospace applications include polyetherimide (PEI) [4,10], polyetherketoneketone (PEKK) [11], nylon or polyamide [12], polycarbonate (PC) [13,14], and polysulfone (PSU).

The suitable 3D polymer material is chosen depending on the particular requirements of the structural component, as different materials offer varying levels of strength, temperature resistance, and other properties. Ultem 9085 (PEI) [15], for example, is a high-performance thermoplastic known for its excellent strength and resistance to high temperatures. It is often used in the aerospace industry to create parts that must withstand extreme conditions (including high resistance to fire), such as engine components and interior cabin parts. Antero 800NA (PEKK) [16] is another high-performance polymer that offers excellent mechanical properties, chemical resistance, and high-temperature resistance and is used in aerospace for components like connectors, brackets, and structural parts. PC is valued for its impact resistance and optical clarity and is used for aircraft windows, displays, and structural components. PSU is a high-temperature thermoplastic known for its toughness and stability and is used for engine components, air ducts, and electrical connectors in aerospace applications. Additionally, ongoing research and development in materials science continue to expand the range of materials available for aerospace 3D printing.

If compared, Ultem 9085 and Antero 800NA are high-performance thermoplastic materials used in aerospace and other advanced applications with fundamental properties [17]. Ultem 9085 is a PEI-based material with food safety and bio-compatibility certification, high heat and chemical resistance, tensile strength, outstanding strength, and thermal stability. Antero 800 NA is a PEKK-based material with elevated heat and chemical resistance, low outgassing, high dimensional stability, and excellent strength, toughness, and wear-resistant properties. Based on an extensive evaluation of the material’s performance, Boeing has released specification BMS8-444 and added the Antero 800NA material to the Qualified Products List (QPL) to meet applications that could not have been 3D-printed before [18].

Besides the excellent properties of these materials, it should be noted that one of the main features of all polymeric parts produced through FFF is their pronounced anisotropy. This is primarily attributed to the inherent properties of the extruded filaments (traxels), the layer-oriented construction method, and the limited degree of fusion between individual layers [4,19]. These issues result in anisotropy in the flame-retardant [12,20], mechanical (including fatigue) [4,10,13,21,22,23] and thermophysical [4,11,21,24,25] characteristics of 3D-printed polymer parts, particularly in the direction perpendicular to the layering process (XZ). Moreover, because of voids and porosity inherent to the FFF printing process, polymer samples fabricated using FFF typically exhibit reduced mechanical strength and possess inferior overall mechanical properties when compared to components produced using conventional manufacturing methods like compression and injection moulding.

This study aimed to directly compare several important physical properties, such as tensile, thermal, and flame-retardant properties, of test samples made from ultra-performance 3D printing materials, polyetherimide (PEI) and polyetherketoneketone (PEKK). Since the samples were printed at different printing orientations, it was possible to evaluate the anisotropy for these properties introduced due to the FFF process, which is essential in fabricating intricately shaped products. To our knowledge, no such or similar research has been published in the literature. The comparison of these properties highlights the advantages and disadvantages of the investigated materials regarding potential applications in the aerospace sector.

## 2. Materials and Methods

### 2.1. Investigated Materials and Test Samples

The investigated materials Ultem 9085 CG (Certified Grade) and Antero 800NA were provided by Stratasys (Eden Prairie, MN, USA) and used at Baltic3D.eu to produce test samples for tensile, thermal, and vertical burn testing from the same batch using the Stratasys F900 machine (Eden Prairie, MN, USA). The infill density was set to 100% (solid) for all samples, and the samples were printed without a border at a raster width of 0.508 mm, slice height of 0.254 mm, contour to the raster air gap, and raster to raster air gap of 0 mm. Printing with borders typically refers to a printing setting that adds a layer (-s) around the printed structure. Adding borders helps prevent the content from getting clipped or cut off and improves the visual appearance of the 3D-printed structure. Nevertheless, to perform tensile, thermal and vertical burn tests, which are formulated by standards ISO 527-1 [26], ASTM E831-19 [27], and FAR/CS 25.853, Appendix F, Part I [28], accordingly, the test samples are required to be without border because the border can affect the properties of the samples printed in different orientations differently. Therefore, printing without a border is preferred to eliminate these issues and compare the results more precisely.

**Figure 1:**
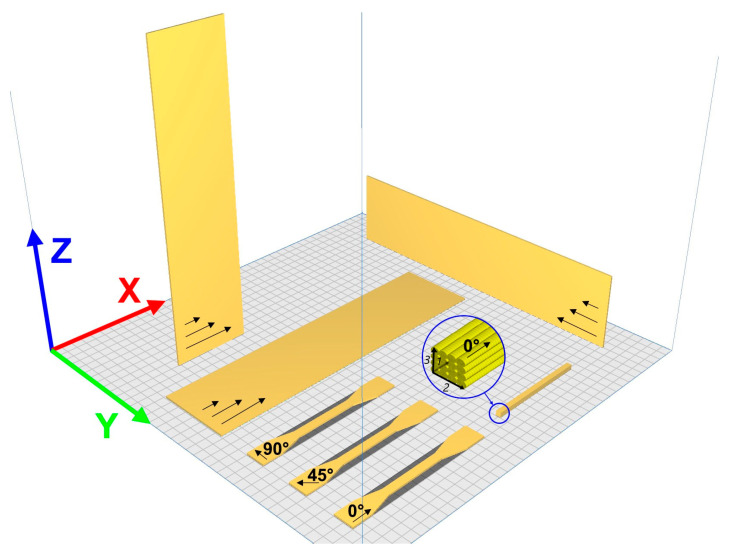
Scheme of the coordinate systems (X-Y-Z and *1-2-3*) introduced and all specimen types.

The printing direction in manufacturing processes can significantly impact the desired properties of the final product. The importance of printing direction lies in its influence on mechanical, thermal, and structural characteristics due to anisotropic behaviour, usually characteristic of 3D-printed parts. Therefore, understanding and optimizing the printing direction can help minimize or leverage this anisotropy based on the application requirements. Thus, two coordinate systems were introduced to define the direction of the extruded filaments concerning printed sample geometry. The first one is the X-Y-Z system characterizing the printing direction, for which the X and Y axes correspond to the bed plane of the printer, while the Z axis is perpendicular to them. The second one is a 1-2-3 system defined as the extruded filament (material) system with the *1*-axis (raster orientation) directed along the filaments, *2*-axis transverse to it, and *3*-axis normal to the 1- and 2-axes plane. The scheme of these coordinate systems and test samples is provided in Figure 1.

The dogbone specimens for tensile tests were printed with sizes according to standard ISO 527-1 (Type 1A). All test samples were printed in the XY plane along the X axis at different raster angles, 0, 45, and 90°, to the sample length. All of the rest of the printing parameters were kept constant. Moreover, virgin filament and stripes printed for the calibration of the 3D printing machine were also tested for comparative purposes.

**Figure 2:**
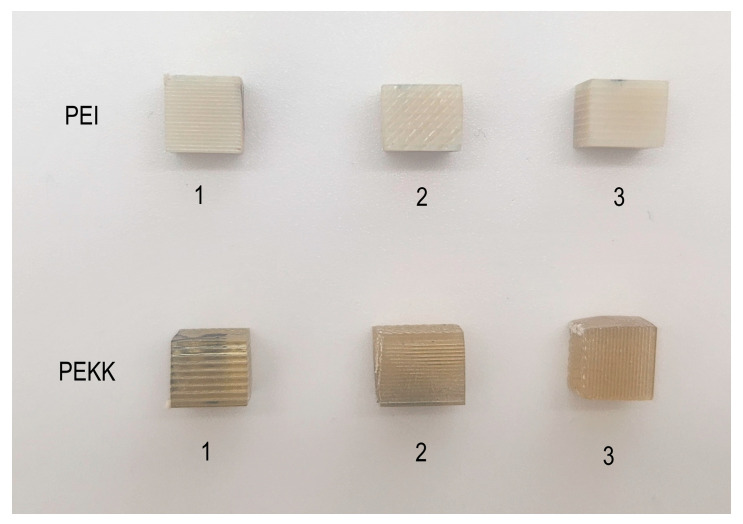
PEI (**top**) and PEKK (**bottom**) test samples were printed for the TMA.

Three sets of samples were prepared to assess the thermal properties of PEI and PEKK. The first set, with dimensions 7 × 7 × 3 mm, was obtained from dogbone samples and employed to determine the softening temperature via penetration measurements through thermomechanical analysis (TMA). Subsequently, the second and third sets, with dimensions of approx. 5 × 5 × 5 mm (shown in Figure 2) were cut from a rectangular bar of 5 × 5 × 100 mm for the assessment of the coefficient of linear thermal expansion (CLTE) and irreversible thermal strain (ITS). The thermal tests were performed in three primary measurement directions, axial (*1*), transverse (*2*), and through-thickness (*3*), for each of the sample sets. The axial measurement direction coincided with the filament direction, while the transverse and through-thickness directions were mutually perpendicular to the axial direction.

For the vertical burn tests, the samples were printed with dimensions 75 × 305 mm according to test criteria and procedures for showing compliance with CS 25.853, 25.855, and 25.869 [29]. The dimensions and printing parameters for both materials studied are provided in Table 1. The test samples were printed in three directions: X, Y, and Z, as presented in Figure 1. At least three test samples were evaluated for each sample configuration (X, Y, and Z), and the reported values represent their arithmetic mean.

### 2.2. Tensile Tests

Quasi-static tensile tests were performed for all test specimens with different filament orientations (0, 45, and 90°) using a Zwick 2.5 testing machine (Zwick Roell Group, Ulm, Germany) at a strain rate of 5 mm/min and room temperature of 20 °C.

The tensile strength was assumed at the maximal value of stress the specimen can withstand, while the elastic modulus was evaluated from the stress–strain diagram as a slope of a secant line for the strain interval from 0.05 to 0.25%. Five test samples per filament orientation, filament pieces, and strips were tested, and the values corresponded to their arithmetic mean value.

The fracture surfaces of the dogbone test samples of PEI and PEKK were examined using a Canon EOS 40D digital SLR camera (Canon Inc., Shimomaruko, Japan). The isometric photos of the fractured surfaces were made for both materials’ samples printed at 0, 45, and 90°.

### 2.3. Thermomechanical Analysis

The softening temperature (*T*_S_) was evaluated using penetration measurements via thermomechanical analysis (TMA) performed with Mettler-Toledo TMA/SDTA 841e (Greifensee, Switzerland). The penetration of a ball-point probe into the specimen was registered as a function of temperature in the range of 30–240 °C at a heating rate of 2 °C/min and an applied load of 1 N.

**Figure 3:**
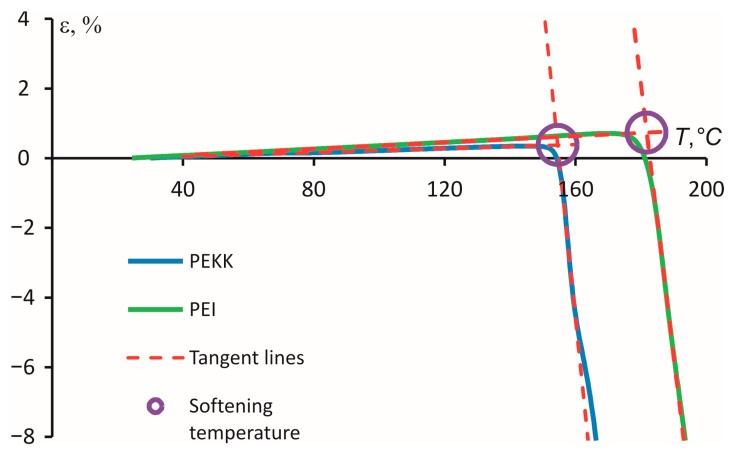
Estimating the softening temperature via a TMA penetration test from deformation as a function of the temperature.

The curves in Figure 3 show the dependence of the relative penetration depth of the probe concerning the initial sample thickness on the temperature. The point of intersection of two tangent lines corresponds to the *T*_S_. The error evaluation shows that the accuracy of determining *T*_S_ is ±1 °C.

**Figure 4:**
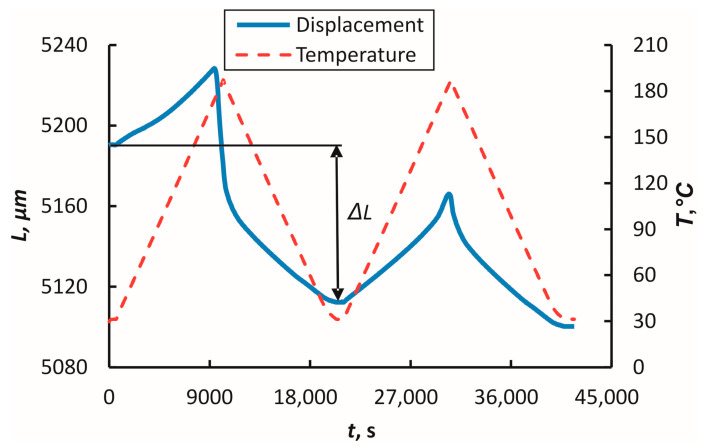
Typical dependence of specimen length and temperature as a function of time during the TMA test for the PEI specimen.

To examine the coefficient of linear thermal expansion (CLTE, α) and irreversible thermal strain (ITS, ε_ITS_), the samples were exposed to two heating/cooling cycles at a temperature range from 30 °C to *T*_S_ at a heating rate of 1 °C/min according to [27]. During TMA, the dependencies of displacement on time along the material coordinate system *1*-*2*-*3* were obtained and used to evaluate the ITS and CLTE. The ITS values were obtained from the first heating/cooling cycle, as shown in Figure 4.

The ITS was calculated from the displacement values:

(1) $$\varepsilon_{ITS}(\%)=\frac{\Delta L}{L_{0}}\times 100,$$

where ∆*L* = *L*_1_ − *L*_0_ is the change in the dimensions of the specimen, and *L*_0_ is the initial length of the specimen (see Figure 4).

The CLTE values were obtained from the second heating/cooling cycle because thermal deformation was observed during the first heating/cooling cycle (Figure 4). The coefficient of thermal expansion values was evaluated using the dependencies of deformation on temperature and Equation (2). The CLTE values (*α*) were evaluated as the derivative of the displacement by temperature:

(2) $$\alpha =\frac{1}{L_{0}}\frac{dL}{dT}.$$

### 2.4. Vertical Burn Tests

Vertical burn tests were conducted at Lantal Textiles AG (Langenthal, Switzerland) following aviation standards FAR/CS 25.853, Appendix F, Part I [28]. A MarlinEngineering (Bellingham, WA, USA) ME 1000-3 flammability chamber was used for all tests. Before the tests, all specimens were conditioned at 22.5 °C and 50% relative humidity (RH). During these tests, the material’s resistance to a flame, maintained at a temperature of 840 °C, was evaluated following the requirements of the 60-second vertical burn test (V60).

**Figure 5:**
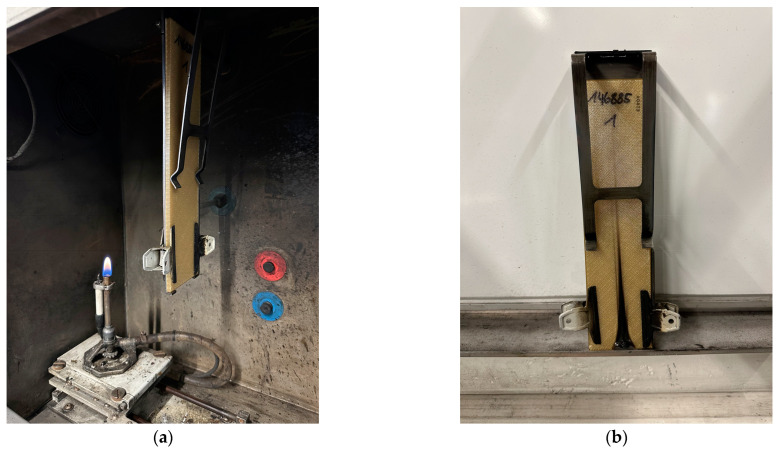
An experimental setup was used for vertical burn tests and PEKK samples (**a**) before and (**b**) after the test.

The samples were hung vertically in these tests, with a burner positioned 19 mm below the lower leading edge for 60 s. Subsequently, the flame was extinguished. The burner was configured to generate a 23 mm core flame and a 38 mm outer flame for all of the tests. Key parameters assessed in the test include ignition time, burn length, flame time, and drip flame time, as previously reported in [12,20]. The experimental setup and PEKK sample before and after the test are shown in Figure 5.

To successfully pass the 60-s vertical burn test, specific criteria must be met for the samples: the average flame time (time until self-extinguishing) must not exceed 15 s; the average drip extinguishing time must not exceed 3 s; the average burn length should not exceed 150 mm [29].

## 3. Results and Discussion

### 3.1. Tensile Properties

**Figure 6:**
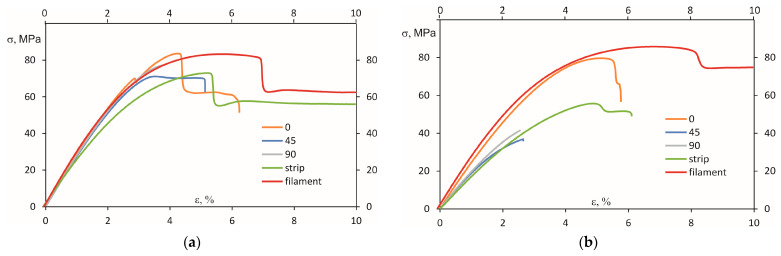
Representative stress–strain curves of (**a**) PEI and (**b**) PEKK at different raster angles and for the filaments and strips.

Experimentally measured stress–strain curves of PEI and PEKK materials are shown in Figure 6. From these curves, it could be observed that the effect of filament orientation on the mechanical behaviour is substantial for PEI but not so essential for PEKK. The graphs were intentionally cut to 10% for the strain axis to focus on the initial stage of the deformation.

First, the highest mechanical performance was obtained for both materials when the raster angle was 0° (along the length). According to Figure 6a, for PEI with the raster angle of 45 and 90°, the resulting stress–strain curves were positioned much lower, indicating the appearance of possible defects. For PEKK (Figure 6b), no significant filament orientation effects were observed, indicating a similar distribution of voids inside the material.

Secondly, it can be observed from Figure 6 that the stress–strain curves of the virgin filaments are higher for both materials than those for the strips. It could indicate internal defects such as voids and an uneven diameter of the strips due to processing. However, it should be mentioned that for PEKK, the difference between the stress–strain curves of the filament and strips was lower than for PEI. Moreover, the maximal deformations observed in Figure 6 were the highest for the filaments and strips made of PEKK, revealing relatively high plastic deformations.

**Figure 7:**
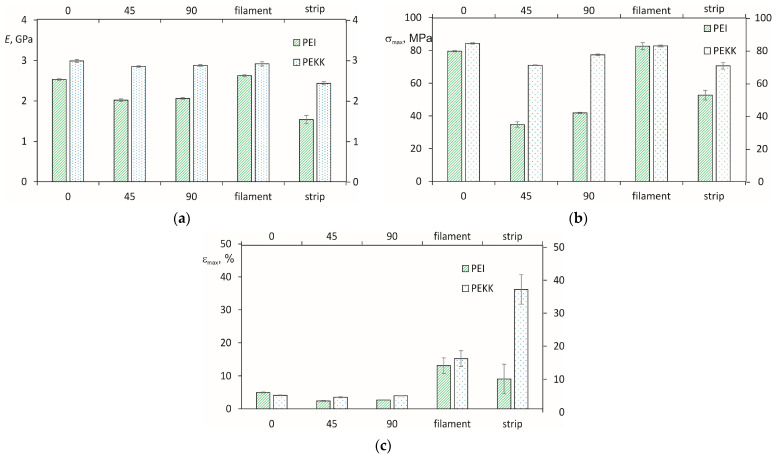
(**a**) Elastic modulus, (**b**) tensile strength, and (**c**) maximal elongation of PEI and PEKK.

To examine the effect of filament orientation on the mechanical properties, the elastic modulus, tensile strength, and maximal elongation were plotted for the materials investigated (Figure 7). From this figure, it is clear that the elastic modulus and strength of PEKK for all tested sample configurations are significantly better if compared with PEI. For the raster angle of 0°, the elastic modulus and strength of PEKK were higher by 18.2% and 6.0% compared with PEI. Interestingly, the mechanical characteristics of these materials for filaments were very close—the elastic modulus of PEKK was higher by 11.1%, but the strength was almost the same if compared with PEI. A more prominent difference in these mechanical characteristics was revealed for the raster angle of 45 and 90° and strips. For instance, for the raster angle of 45°, the elastic modulus and tensile strength of PEKK were higher by 41.1 and 104.4%, respectively, compared to PEI.

According to Figure 7, the maximal elongation of PEI and PEKK was comparable for the dogbone samples and filaments but was very different for the strips. For them, the maximal elongation of PEKK was higher by approx. 300% if compared to PEI. Also, dogbone samples with the raster angle of 45 and 90° and filament PEKK revealed a higher value of maximal elongation, which is entirely in line with the characteristics provided in the materials’ datasheets published by Stratasys [15,16]. Thus, for PEI, the maximal elongation for the samples consisted of 10 layers and, printed along a sample length with 0.254 mm layer heights, was 5.4 ± 0.5%, but for PEKK, it was 6.1 ± 0.9%. It should be noted that due to the internal voids characteristic of 3D-printed parts produced using FFF the mechanical characteristics of the base materials, PEI and PEKK, were higher than the results obtained for the samples printed at different orientations, filaments, extruded strips, (Figure 7) and the data provided for both material datasheets [15,16]. For PEI, the tensile strength reported in the literature [30,31] was 102.5–105.2 MPa, the elastic modulus was 2.93–3.01 GPa, and the maximal deformation was 18%. For PEKK, the reported results [32,33] were higher than the obtained ones; the tensile strength was 117 MPa, but the elastic modulus was 4.4 GPa, accordingly.

**Figure 8:**
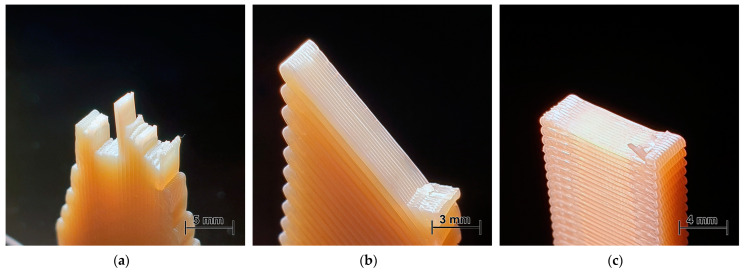
Photos of fractured dogbone samples of PEI samples printed at (**a**) 0, (**b**) 45, and (**c**) 90°.

**Figure 9:**
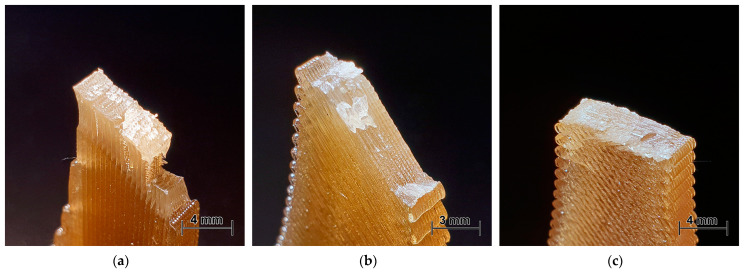
Photos of fractured dogbone samples of PEKK samples printed at (**a**) 0, (**b**) 45, and (**c**) 90°.

The fracture surfaces of the dogbone samples of PEI and PEKK were analysed to reveal the peculiarities of the failure modes. The isometric photos of fractured test samples after the tensile tests of PEI and PEKK printed at 0, 45, and 90° are provided in Figure 8 and Figure 9, respectively. Upon visual analysis, it can be observed that cracking predominantly occurred and propagated along the raster orientation. Thus, the failure mechanism was affected by weak interlayer bonding, which is attributed to the volumetric shrinkage during polymer solidification and cooling [34]. Comparing Figure 8 and Figure 9, it can be concluded that the interlayer bonding (adhesion) was lower for PEI, revealing smoothly separated layers without rough surfaces, as was observed for PEKK. Generally, samples printed at a 0° angle experienced failure primarily through fibre breakage or pullout at high strains, revealing noticeable necking and thinning across the entire gauge length (Figure 8a and Figure 9a). Meanwhile, the samples printed at 45 and 90° exhibited visible fractures along the interlayer bonding at 45 and 90° angles, respectively. These samples mostly fractured immediately upon reaching ultimate strength or had a limited necking phase, as shown in the stress–strain curves for PEI and PEKK in Figure 6.

The effects of several printing parameters (air gap, raster width and angle, contour number and width) were comprehensively studied for the tensile properties of PEI samples in [23]. It was found that the raster angle had the most significant effect on the tensile strength and strain. Thus, the samples printed at the raster angle of 0° exhibited greater strength compared to those with a 90° raster angle. This superiority was described by the production method employed for samples with a 0° raster angle, aligning filaments longitudinally along the sample’s length. Such filament alignment along the direction of tensile load application enhanced the material’s resistance to applied tensile forces, consequently improving its tensile properties. Similar results were obtained for tensile and flexural properties of PEI printed at different raster angles (0, ±45, and 90°) and printing orientations (X, Y, and Z) [35]. For all printing directions, both tensile and flexural properties of PEI samples printed at a raster angle of 0° were higher if compared with a 90° raster angle. As described in [35], for the bending test, the raster orientation is parallel to the load applied, facilitating force to separate the filaments and cause fracturing at their interface.

The influence of printing orientation (X, Y, and Z) and raster angle (0, 45, and 90°) on the tensile and fatigue properties was analysed for PEI and PEKK [36]. It was shown that PEKK samples were characterized with better tensile properties for all three printed orientations than PEI samples. Also, fatigue resistance was higher for PEKK if compared with PEI results, revealing higher monotonic strength values and lower exponent values to fit the fatigue data.

Moreover, the degradation of tensile properties due to moisture absorption was studied for twelve different filaments, including PEI and PEKK [3]. According to the results obtained, both materials were classified as having a moderate (5–9%) sensitivity to water by their reduction in mechanical properties, while the maximal moisture sorption capacity and swelling coefficient of PEI were almost twice as high as for PEKK. These results and superior mechanical performance make PEKK a more environmentally stable and durable material than PEI.

### 3.2. Thermal Properties

During the penetration test, the softening temperature values for samples were obtained. For PEI, the softening temperature value reached 155 °C and 182 °C for PEKK.

**Figure 10:**
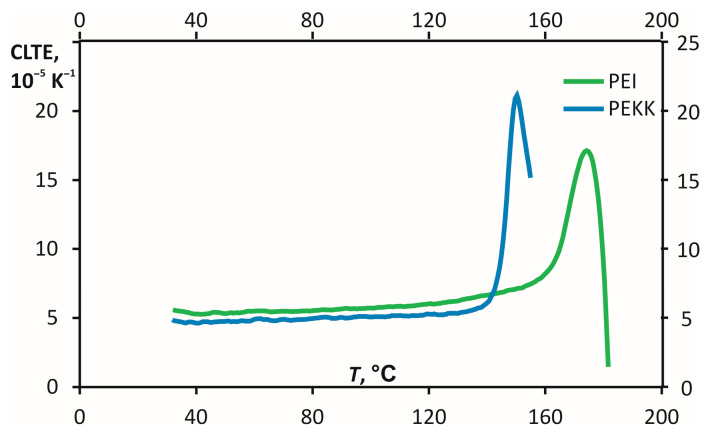
Dependences of CLTE of the samples as a function of the temperature of PEI and PEKK for the axial measurement direction (the materials are indicated on the graph).

The dependencies of the CLTE on the temperature in the X direction in the second heating/cooling cycle were obtained using TMA (Figure 10). As shown in Figure 10, the CLTE slowly grows at increasing temperatures until 140 °C for PEKK and 160 °C for PEI. Further, the CLTE curves rapidly grow and achieve the maximum value at the near-softening temperature and sharply decrease at temperatures exceeding the softening temperature because of the loss of their dimension stability (Figure 10).

**Figure 11:**
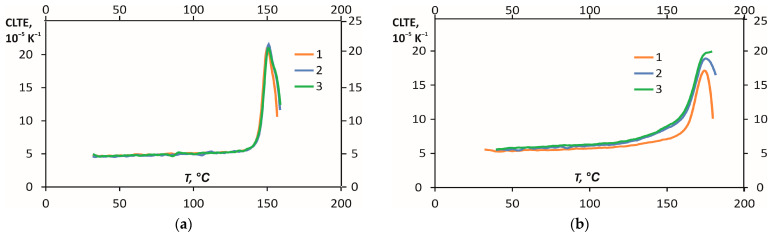
CLTE values of PEKK (**a**) and PEI (**b**) in the measurement directions *1*, *2*, and *3* (as defined in Figure 1).

Previously, the values of CLTE in the three measurement directions and different temperature ranges were obtained [11]. The values of CLTE for PEKK were lower than PEI until the temperature range from 150 to 165 °C and sharply increased three to four times the temperature range from 150 to 165 °C. Therefore, it can be concluded that PEI is more thermally stable than PEKK. The materials have almost the same CLTE values, but PEKK has isotropic behaviour for the thermal expansion, as seen from the CLTE results in three measurement directions *1-2-3* (Figure 1), shown in Figure 11. Thus, estimating the degree of anisotropy for CLTE is possible, defined as the ratio of the maximal values for two components in different directions (*1-2*, *2-3*, *1-3*). PEKK’s degree of anisotropy was about 1.02, and PEI was about 1.17.

The observed anisotropy in the thermal expansion between PEKK and PEI can be attributed to the molecular structure and crystallinity differences. PEKK is a semicrystalline polymer with a highly ordered molecular structure [37]. Crystalline regions in polymers tend to have lower thermal expansion than amorphous regions. Thus, the arrangement of polymer chains in the crystalline phase contributes to the anisotropic thermal behaviour. PEI is amorphous and lacks a well-defined crystalline structure. Amorphous polymers generally exhibit higher thermal expansion because their molecular chains are not as ordered [38]. Moreover, the degree of crystallinity in PEKK influences its thermal expansion properties. Therefore, higher crystallinity often leads to lower thermal expansion in the direction of the polymer chains.

**Figure 12:**
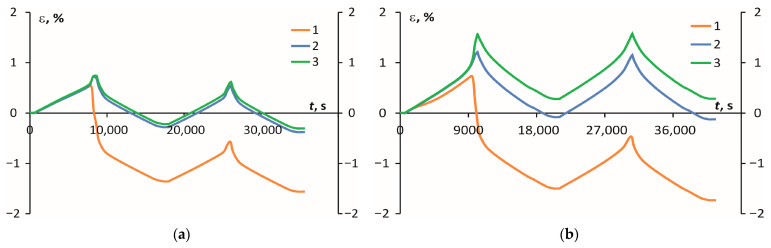
The curves of deformation (ε) dependence on time (*t*) for PEKK (**a**) and PEI (**b**) in the measurement directions *1*, *2*, and *3* (as defined in Figure 1).

The values of the ITS were calculated in the first heating/cooling cycle. During the investigation, the dependencies of deformation on time were obtained and are provided in Figure 12. Thermal deformation was observed during the relaxation process on the first heating/cooling cycle. After that, the materials exhibited thermally stable behaviour, as seen in Figure 12. In the case of PEKK, the material shrinkage in all three material coordinate system directions was −1.36% (*1*), −0.28% (*2*), and −0.22% (*3*). For PEI in directions *1* and *2*, there was material shrinkage, by −1.50% and −0.08%, but in *3* it expanded by 0.28% (*3*), respectively. Both materials had significant thermal deformation in measurement direction *1* compared to the other directions. Notably, the samples shrunk in direction 1 for both materials and expanded in direction *3*.

The main factors contributing to the shrinkage in semicrystalline polymers during the cooling process include the enhancement of crystallization, which is facilitated by the parallel alignment of macromolecules, resulting in the preferential orientation of the crystalline lamellae due to flow-induced effects [39] and the development of internal stresses due to the freezing-in of crystallite and molecular orientations [38,40]. Therefore, the lower shrinkage rate of PEKK (see Figure 12) could be explained by the re-organization of macromolecular chains to crystallites or the parallelization of macromolecules in a semicrystalline PEKK that occurs during the cooling process and does not occur in an amorphous PEI.

The inherent anisotropy in the FFF manufacturing process was also demonstrated through an analysis of the coefficient of thermal expansion for different 3D printing materials, resulting in diminished reinforcement properties when materials are vertically printed (in the Z printing direction), primarily due to inadequate adhesion between layers during the deposition of molten polymeric layers [37,41]. The results obtained for CLTE of PEI printed in flat print orientation and tested in axial (*1*), transverse (*2*), and trough-thickness (*3*) directions also revealed the anisotropy of the results with the highest values obtained for direction *3* and the lowest one for the direction *1* [21].

### 3.3. Fire-Retardant Properties

**Figure 13:**
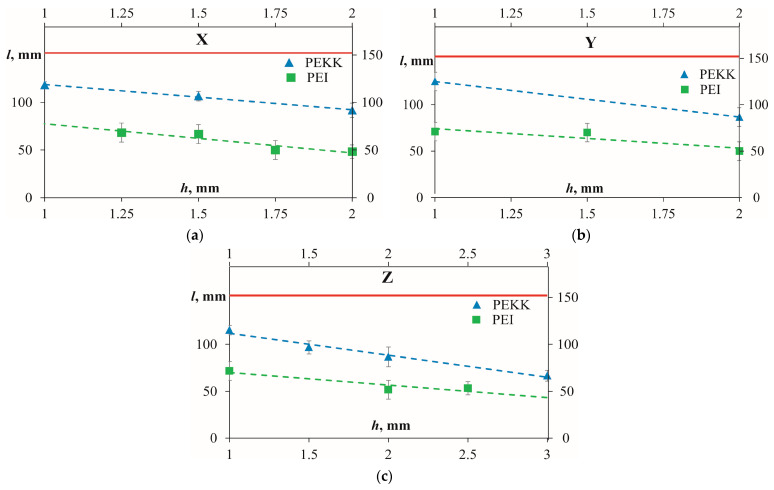
Vertical burn length (*l*) vs. thickness (*h*) for PEKK and PEI samples (as indicated on the graph) printed in (**a**) X, (**b**) Y, and (**c**) Z printing directions. The solid red line is the threshold to pass the test; the dashed lines are linear approximations.

Vertical burn tests used in the current work are fire resistance or flammability tests conducted to determine how a material reacts when subjected to a controlled open flame in a vertical orientation. The results obtained for the burn length for PEI and PEKK samples printed at an infill density of 100% but different thicknesses and printing orientations X, Y, and Z are provided in Figure 13. It can be noticed that in all printing orientations, PEKK samples had significantly larger burn lengths in comparison with PEI samples. Thus, according to Figure 13, both materials passed the minimal requirement, which did not exceed 150 mm for the burn length after an ignition time of 60 s, but based on the results obtained, PEI for all thicknesses of the samples was more efficient in fire resistance if compared with PEKK. The excellent fire-resistant properties of PEI and PEKK, which satisfy the fire safety regulatory standard EASA CS 25 [28], are discussed in [20,42,43,44]. The decrease in the burn length as a function of the thickness of the sample can be described by the more considerable amount of material contained in the samples and, consequently, the smaller burn length [43].

**Figure 14:**
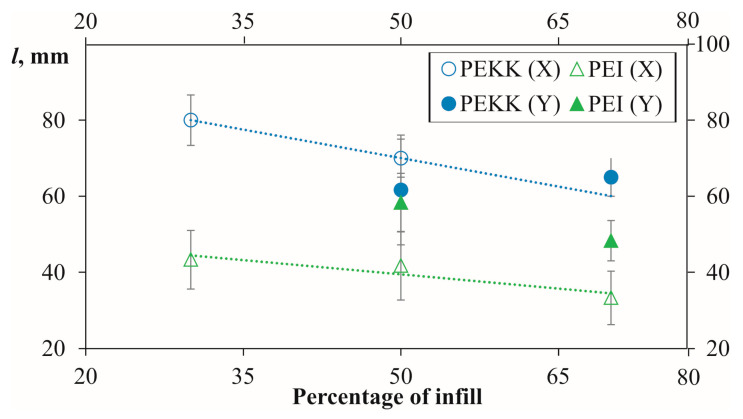
Vertical burn length (*l*) for PEKK and PEI samples printed in directions X and Y as a function of infill percentage (indicated on the graph) at 5 mm thickness and two solid layers.

Furthermore, the results provided in Figure 14 compare burn lengths for PEKK and PEI across various infill percentages, maintaining a constant thickness of 5 mm and featuring two solid layers on both sides. Figure 14 reveals that the burn length is notably higher in the 30% infill samples than in the 50 and 70% infill samples. The general trend of burn length decreasing with an increase in infill percentage from 30 to 70% is evident from a material content perspective [20].

Additionally, it is essential to note that the range of experimental errors was significantly more extensive in all cases, suggesting a potential lack of control in the metrics used for data collection. Also, it can be noted that the results obtained for the X and Y build directions were close for PEKK and more distant for PEI. Similar results were obtained for these materials’ tensile and thermal properties, proving less anisotropy for the PEKK samples. Therefore, a thorough comprehension of the relationship between structure and performance is crucial for fire-retardant polymeric composites. This understanding facilitates the development of advanced fire-retardant polymeric materials with optimized performance characteristics [45,46].

In general, the printing orientation of a 3D-printed part can affect its fire-retardant properties due to the anisotropic nature of the printing process characteristic of FFF and the resulting material characteristics. The following factors related to raster orientation can affect the fire-retardant properties of 3D-printed parts: adhesion between layers, material flow and density, and internal void content [42]. For some raster orientations, there may be better layer-to-layer bonding, creating a more robust structure that can withstand fire exposure. Moreover, the flow of the printing material during the printing process can influence the density and structure of the final object. Thus, varying the printing orientation may affect the arrangement of the material, potentially influencing its ability to resist or slow down the spread of fire [42]. Finally, the printing orientation can affect the presence of internal voids or trapped air within the printed object. These voids can act as insulators, influencing the material’s ability to conduct heat and contributing to its fire-retardant properties [47].

Still, the results showed that PEI was more efficient in fire resistance than PEKK. The only direct comparison of these materials for flame-retardant properties was found in the literature [48]. Based on the microscale combustion calorimetry results [48], PEI and PEKK were classified as superior materials regarding flammability properties. While PEI, in comparison with PEKK, showed lower heat release capacity and peak heat release rate, it had a slightly lower peak temperature. Therefore, considering significant data scattering for PEI, it was concluded that both materials can be assumed to be similar in flammability performance.

The intrinsic flame-retardant parameters of PEI (Ultem 9085) and PEKK (Antero 800 NA) can be compared by using material datasheets provided by Stratasys [15,16]. After 60-s ignition, according to JAR/FAR/CS 25.853 (a), Appendix F, Part A, the average burn length of PEI is 10.16 and 15.24 mm in flat (XY) and on-edge (XZ) print orientations, accordingly [15]. For PEKK, much higher results for the average burn length are reported—62.23 for flat and 72.64 mm for on-edge print orientations [16]. These results can be used for comparative purposes since the datasheets do not provide the thickness, infill density and raster orientation. Still, the results obtained correlate well with the information from the datasheet, revealing lower values for the burn length of PEI compared with PEKK.

## 4. Conclusions

It was experimentally confirmed that the tensile characteristics (elastic modulus and tensile strength) of PEKK exceeded PEI’s characteristics, especially when the raster angle was 45 and 90°. A negligible difference in tensile characteristics was obtained for a raster angle of 0° and virgin filaments. It indicated the possible discrepancy of void distribution in PEI at different filament orientations, which could be further studied.

For PEKK, no essential effect of raster angle on tensile properties was revealed. The maximal elongation of PEI and PEKK was comparable for the dogbone samples and filaments but was very different for the strips, revealing high plastic deformations for PEKK. Higher values of all tensile characteristics corresponded well to the material properties provided by Stratasys and published in the literature.

Based on the results of thermomechanical analysis, PEI had a higher softening temperature (140 °C and 160 °C for PEKK and PEI, accordingly) and, therefore, was more thermally stable than PEKK. Both materials had significant thermal deformation in measurement direction *1* compared to the other directions.

Similarly, the results obtained for the burn length showed that PEI was more efficient in fire resistance than PEKK at different thicknesses and infill percentages. Definitely, PEKK had better isotropic behaviour than PEI for mechanical performance, thermal expansion, and fire-resistant properties. The isotropy in all of these properties among 3D printing materials is essential in fabricating intricately shaped products. Moreover, it confers notable advantages over traditional machining methods, leading to accelerated production, reduced material wastage, and lower energy consumption.

Nevertheless, all test samples (also at low infill percentage) for both materials have passed the flammability aviation requirements as per EASA CS 25 for the materials used in the compartment interiors and cargo or baggage compartment. Thus, these materials and FFF are suitable for aircraft interior compartments. Low infill density values for the 3D printed structures can contribute to the reduction of the aeroplanes’ overall weight, fuel consumption, and CO_2_ release.

## Figures and Tables

**Table 1:** Dimensions and printing parameters for PEI and PEKK samples prepared for the vertical burn tests.

Thickness, mm	Print Direction	Number of Solid Layers	Infill Percentage, %
1	X, Y, Z	1	100
1.5	X, Z	1	100
2	X, Y, Z	1	100
5	X, Y	2	30, 50, 70

## Data Availability

The data presented in this study are available on request from the corresponding author.

## References

[1] Singh S., Singh G., Prakash C., Ramakrishna S. (2020). Current status and future directions of fused filament fabrication. J. Manuf. Process..

[2] Kumar S., Singh R., Singh T.P., Batish A. (2020). Fused filament fabrication: A comprehensive review. J. Thermoplast. Compos. Mater..

[3] Aniskevich A., Bulderberga O., Stankevics L. (2023). Moisture sorption and degradation of polymer filaments used in 3D printing. Polymers.

[4] Glaskova-Kuzmina T., Dejus D., Jātnieks J., Aniskevich A., Sevcenko J., Sarakovskis A., Zolotarjovs A. (2023). Effect of Post-Printing Cooling Conditions on the Properties of ULTEM Printed Parts. Polymers.

[5] Diniță A., Neacșa A., Portoacă A.I., Tănase M., Ilinca C.N., Ramadan I.N. (2023). Additive Manufacturing Post-Processing Treatments, a Review with Emphasis on Mechanical Characteristics. Materials.

[6] Jayawardane H., Davies I.J., Gamage J.R., John M., Biswas W.K. (2023). Sustainability perspectives—A review of additive and subtractive manufacturing. Smart Sustain. Manuf. Syst..

[7] Kanishka K., Acherjee B. (2023). A systematic review of additive manufacturing-based remanufacturing techniques for component repair and restoration. J. Manuf. Process..

[8] Vidakis N., Petousis M., Velidakis E., Mountakis N., Fischer-Griffiths P.E., Grammatikos S.A., Tzounis L. (2022). Fused filament fabrication 3D printed polypropylene/ alumina nanocomposites: Effect of filler loading on the mechanical reinforcement. Polym. Test..

[9] Kobenko S., Dejus D., Jātnieks J., Pazars D., Glaskova-Kuzmina T. (2022). Structural integrity of the aircraft interior spare parts produced by additive manufacturing. Polymers.

[10] Glaskova-Kuzmina T., Dejus D., Jātnieks J., Kruuv P.-P., Zolotarjovs A., Einbergs E., Vanags E. (2023). Effect of printing direction and post-printing conditions on bending properties of ULTEM 9085. J. Compos. Sci..

[11] Bute I., Tarasovs S., Vidinejevs S., Vevere L., Sevcenko J., Aniskevich A. (2023). Thermal properties of 3D printed products from the most common polymers. Int. J. Adv. Manuf. Technol..

[12] Glaskova-Kuzmina T., Dejus D., Jātnieks J., Kruuv P.-P., Lancere L., Kobenko S., Sarakovskis A., Zolotarjovs A. (2022). Flame-retardant and tensile properties of the polyamide-12 processed by selective laser sintering. J. Compos. Sci..

[13] Bakhtiari H., Aamir M., Tolouei-Rad M. (2023). Effect of 3D Printing Parameters on the Fatigue Properties of Parts Manufactured by Fused Filament Fabrication: A Review. Appl. Sci..

[14] Harris M., Potgieter J., Archer R., Arif K.M. (2019). Effect of Material and Process Specific Factors on the Strength of Printed Parts in Fused Filament Fabrication: A Review of Recent Developments. Materials.

[15] Stratasys, ULTEM 9085 Production-Grade Thermoplastic for Fortus 3D Printers. https://www.stratasys.com/en/materials/materials-catalog/fdm-materials/ultem-9085/.

[16] Stratasys, Antero 800NA, FDM PEKK Thermoplastic, High-Performance PEKK Polymer. https://www.stratasys.com/en/materials/materials-catalog/fdm-materials/antero-800na/.

[17] Stratasys, Stratasys FDM 3D Printers and Materials. FDM Reliable. Repeatable. Exceptional. https://www.stratasys.com/contentassets/1c85ac2637f341a9bde91be45fe29b6d/br_fdm_systemsoverview_0322a.pdf?v=49ae11.

[18] Aeroflap, Boeing Qualifies 3D Printing Material to be Used in Parts of the Company’s Aircraft. https://www.aeroflap.com.br/en/boeing-qualifies-3d-printing-material-to-be-used-in-parts-of-the-company%27s-aircraft/?amp=1.

[19] Tofangchi A., Han P., Izquierdo J., Iyengar A., Hsu K. (2019). Effect of ultrasonic vibration on interlayer adhesion in fused filament fabrication 3D printed ABS. Polymers.

[20] Lv Y., Dejus D., Kobenko S., Singamneni S., Glaskova-Kuzmina T. (2022). Evaluation of the fire-retardancy of ULTEM 9085 polymer composites processed by fused deposition modelling. Mater. Sci..

[21] Zaldivar R.J., Witkin D.B., McLouth T., Patel D.N., Schmitt K., Nokes J.P. (2017). Influence of processing and orientation print effects on the mechanical and thermal behavior of 3D-Printed ULTEM^®^ 9085 Material. Addit. Manuf..

[22] Ngo T.D., Kashani A., Imbalzano G., Nguyen K.T.Q., Hui D. (2018). Additive manufacturing (3D printing): A review of materials, methods, applications and challenges. Compos. Part B.

[23] Gebisa A.W., Lemu H.G. (2017). Influence of 3D printing FDM process parameters on tensile property of ULTEM 9085. Procedia Manuf..

[24] Padovano E., Galfione M., Concialdi P., Lucco G., Badini C. (2020). Mechanical and thermal behavior of Ultem^®^9085 fabricated by fused-deposition modeling. Appl. Sci..

[25] Blanco I. (2022). A brief review of the applications of selected thermal analysis methods to 3D printing. Thermo.

[26] ISO 527-1; Plastics—Determination of Tensile Properties—Part 1: General Principles. https://www.iso.org/standard/75824.html.

[27] (2012). Standard Test Method for Linear Thermal Expansion of Solid Materials by Thermomechanical Analysis.

[28] European Union Aviation Safety Agency, EASA CS 25.853 Appendix F Part I. https://www.easa.europa.eu/en/document-library/easy-access-rules/online-publications/easy-access-rules-large-aeroplanes-cs-25?page=68.

[29] Federal Aviation Regulation (FAR) Standard No. 25.853 Fire Test to Aircraft Material: Fire Protection for Compartment Interior. https://www.federalregister.gov/documents/2019/07/03/2019-13646/interior-parts-and-components-fire-protection-for-transport-category-airplanes.

[30] Sun Z., Li Y.-Q., Huang P., Cao H.-J., Zeng W., Li J., Li F., Sun B.-G., Shi H.-Q., Zhou Z.-L. (2021). Temperature-dependent mechanical properties of polyetherimide composites reinforced by graphene oxide-coated short carbon fibers. Compos. Struct..

[31] Pitchan M.K., Bhowmik S., Balachandran M., Abraham M. (2016). Effect of surface functionalization on mechanical properties and decomposition kinetics of high performance polyetherimide/MWCNT nano composites. Compos. Part A Appl. Sci..

[32] Choupin T., Debertrand L., Fayolle B., Régnier G., Paris C., Cinquin J., Brulé B. (2019). Influence of thermal history on the mechanical properties of poly(ether ketone ketone) copolymers. Polym. Cryst..

[33] Ramaswamy K., Modi V., Rao P.S., Martin P.P., McCarthy C.T., O’Higgins R.M. (2023). An investigation of the influence of matrix properties and fibre–matrix interface behaviour on the mechanical performance of carbon fibre-reinforced PEKK and PEEK composites. Compos. Part A Appl. Sci..

[34] Dolzyk G., Jung S. (2019). Tensile and fatigue analysis of 3D-printed polyethylene terephthalate glycol. J. Fail. Anal. Prev..

[35] Byberg K.I., Gebisa A.W., Lemu H.G. (2018). Mechanical properties of ULTEM 9085 material processed by fused deposition modeling. Polym. Test..

[36] Kaplun B.W., Zhou R., Jones K.W., Dunn M.L., Yakacki C.M. (2020). Influence of orientation on mechanical properties for high-performance fused filament fabricated ultem 9085 and electro-statically dissipative polyetherketoneketone. Addit. Manuf..

[37] Kennedy Z.C., Christ J.F., Fenn M.D., Zhong L., Chouyyok W., Arnold A.M., Denny A.C., Albrecht A.M., Silverstein J.A., Erikson R.L. (2022). Mica filled polyetherketoneketones for material extrusion 3D printing. Addit. Manuf..

[38] Ramakers-van Dorp E., Möginger B., Hausnerova B. (2020). Thermal expansion of semicrystalline polymers: Anisotropic thermal strain and crystallite orientation. Polymer.

[39] Benedetti L., Brulé B., Decreamer N., Evans K.E., Ghita O. (2019). Shrinkage behaviour of semicrystalline polymers in laser sintering: PEKK and PA12. Mater. Des..

[40] Troisi E.M., Caelers H.J.M., Peters G.W.M. (2017). Full characterization of multiphase, multimorphological kinetics in flow-induced crystallization of IPP at elevated pressure. Macromolecules.

[41] De León A.S., Domínguez-Calvo A., Molina S.I. (2019). Materials with enhanced adhesive properties based on acrylonitrile-butadiene-styrene (ABS)/thermoplastic polyurethane (TPU) blends for fused filament fabrication (FFF). Mater. Des..

[42] Lv Y.F., Thomas W., Chalk R., Singamneni S. (2020). Flame retardant polymeric materials for additive manufacturing. Mater. Today Proc..

[43] Das A., Chatham C.A., Fallon J.J., Zawaski C.E., Gilmer E.L., Williams C.B., Bortner M.J. (2020). Current understanding and challenges in high temperature additive manufacturing of engineering thermoplastic polymers. Addit. Manuf..

[44] Vahabi H., Laoutid F., Mehrpouya M., Saeb M.R., Dubois P. (2021). Flame retardant polymer materials: An update and the future for 3D printing developments. Mater. Sci. Eng. R Rep..

[45] Sai T., Ran S., Guo Z., Song P., Fang Z. (2022). Recent advances in fire-retardant carbon-based polymeric nanocomposites through fighting free radicals. SusMat.

[46] Wang Y., Miao Y., Ge B., He Z., Zhu X., Liu S., Li J., Yu L. (2023). Additives enhancing supported amines performance in CO_2_ capture from air. SusMat.

[47] Geoffroy L., Samyn F., Jimenez M., Bourbigot S. (2019). Innovative 3D printed design to conceive highly fire-retardant multi-material. Polym. Degrad. Stab..

[48] Kafi A., Wu H., Langston J., Atak O., Kim H., Kim S., Fahy W.P., Reber R., Misasi J., Bateman S. (2020). Evaluation of additively manufactured ultra performance polymers to use as thermal protection systems for spacecraft. J. Appl. Polym. Sci..

